# An Angiogenic Role for Adrenomedullin in Choroidal Neovascularization

**DOI:** 10.1371/journal.pone.0058096

**Published:** 2013-03-08

**Authors:** Susumu Sakimoto, Hiroyasu Kidoya, Motohiro Kamei, Hisamichi Naito, Daishi Yamakawa, Hirokazu Sakaguchi, Taku Wakabayashi, Kohji Nishida, Nobuyuki Takakura

**Affiliations:** 1 Department of Signal Transduction, Research Institute for Microbial Diseases, Osaka University, Suita, Osaka, Japan; 2 Department of Ophthalmology, Osaka University Graduate School of Medicine, Suita, Osaka, Japan; 3 JST(Japan Science and Technology Agency), CREST, Tokyo, Japan; University of Sydney, Australia

## Abstract

**Purpose:**

Adrenomedullin (ADM) has been shown to take part in physiological and pathological angiogenesis. The purpose of this study was to investigate whether ADM signaling is involved in choroidal neovascularization (CNV) using a mouse model.

**Methods and Results:**

CNV was induced by laser photocoagulation in 8-week-old C57BL/6 mice. ADM mRNA expression significantly increased following treatment, peaking 4 days thereafter. The expression of ADM receptor (ADM-R) components (CRLR, RAMP2 and RAMP 3) was higher in CD31^+^CD45^−^ endothelial cells (ECs) than CD31^−^CD45^−^ non-ECs. Inflammatory stimulation upregulated the expression of ADM not only in cell lines but also in cells in primary cultures of the choroid/retinal pigment epithelium complex. Supernatants from TNFα-treated macrophage cell lines potentiated the proliferation of ECs and this was partially suppressed by an ADM antagonist, ADM (22–52). Intravitreous injection of ADM (22–52) or ADM neutralizing monoclonal antibody (mAb) after laser treatment significantly reduced the size of CNV compared with vehicle-treated controls (p<0.01).

**Conclusions:**

ADM signaling is involved in laser-induced CNV formation, because both an ADM antagonist and ADM mAb significantly inhibited it. Suppression of ADM signaling might be a valuable alternative treatment for CNV associated with age-related macular degeneration.

## Introduction

Aberrant angiogenesis occurs under numerous pathological conditions, such as cancer, rheumatoid arthritis, psoriasis and many ocular diseases. Age-related macular degeneration (AMD) is the leading cause of vision loss in elderly persons in developed countries. Patients with severe vision loss are often affected by wet AMD [1] the central pathologic features of which are recognized as choroidal neovascularization (CNV), induced by a complex pathogenic process whereby new blood vessels are generated from the choriocapillaris beneath the retina. CNV-associated vessels tend to leak and bleed, thereby severely affecting the neural tissue of the macula.

Genetic variation in complement factor genes in AMD patients suggests inflammatory processes as a trigger of drusen formation which is a hallmark of this disease. Moreover, infiltration of inflammatory cells such as macrophages which produce various angiogenic factors could support neovessel formation from the choriocappillaris directly and indirectly [2], [3], [4], [5].

Adrenomedullin (ADM), identified as a potent vasodilator with wide tissue distribution, is a multifunctional 52 amino acid peptide activating heterodimeric receptors composed of a seven transmembrane (7TM) G-protein-coupled receptor (GPCR) calcitonin-receptor-like receptor (CRLR, now known as CL) [6] and receptor activity-modifying proteins (RAMPs) [7]. ADM is also thought to play a critical role in forming blood vessels, with functions including regulation of vascular stability under both physiological and pathological conditions [8]–[10]. Gene targeting analysis in mice showed that global deletion of the ADM gene results in embryonic lethality at E13.5 caused by vascular abnormalities [10].

Expression of ADM is regulated by hypoxia, growth factors and inflammation [6], [8]. Moreover, accumulating evidence for the involvement of ADM in tumor angiogenesis has demonstrated that inhibition of ADM function by neutralizing antibody or the ADM antagonist ADM (22–52) inhibits tumor growth in xenograft models [11], [12], [13]. In vascular endothelial cells (ECs), activation of phosphatidylinositol 3′ kinase (PI3K/Akt), mitogen-activated protein kinase (MAPK) and focal adhesion kinase (p125FAK) plays a role in ADM-induced angiogenesis [8], [14], [15]. The level of ADM expression in tumors correlates with vascular density in patients [16] and ADM-heterozygous knockout mice have reduced neovascularization in a tumor xenograft model [9].

However, it is poorly understood whether ADM could be an effector in other disease models, especially in ocular neovascularization. Therefore, here we investigate whether ADM has a role in proangiogenesis in laser-induced CNV, which is widely accepted as a mammalian AMD model, and have attempted to characterize mechanisms of ADM signaling in CNV formation.

## Materials and Methods

### Animals

All experiments were conducted under the applicable laws and guidelines for the care and use of laboratory animals in the Research Institute for Microbial Diseases, Osaka University, approved by the Animal Experiment Committee of the Research Institute for Microbial Disease, Osaka University.

### Laser-induced CNV and Drug Treatment

Laser photocoagulation (514 nm Argon laser, 150 mW, 50 ms duration, 50 mm spot size; Ultima 2000 SE, Lumenis/Coherent) was performed bilaterally in each 8-week-old wild-type C57BL/6 mouse. A total of 6 laser spots per eye were created in a standard fashion around the optic nerve using a slit lamp delivery system (Carl Zeiss, Germany) and using a cover slip as a contact lens. Only burns that produced a bubble, indicating rupture of the Bruch membrane, were included in the study. Eyes merely touched with a cover slip acted as sham-operated controls. Immediately after laser photocoagulation, mice were randomized into several groups and received intravitreal injections of 1 µl ADM (22–52) (10 µM or 100 µM), ADM (200 µM) (Peptide Institute, Osaka, Japan), SU1498 (10 µM) [17], ADM monoclonal antibody (1.45 mg/ml) (provided by Diagnostic Science Division, Shionogi & Co., Ltd.) or vehicle (PBS). The same treatment was performed 3 days after photocoagulation in the same fashion. Intravitreal injection was performed with the FemtoJet Microinjector System (Eppendorf, Germany) under a high magnification stereomicroscope (Leica M125, Germany). Eyes were enucleated and fixed for immunohistochemistry 7 days after photocoagulation. We used 16 mice and 16 choroidal flatmounts in ADM antagonist experiments, 8 mice and 8 flatmounts in experiments using combination treatment with ADM antagonist and VEGF inhibitor, and 10 mice and 10 flatmounts in ADM mAb experiments.

### Measurement of Laser-induced CNV Size

On day 7 after laser photocoagulation, the sizes of CNV lesions were measured on RPE-choroid flat mounts as described previously [18]. Image J for Windows (NIH, Bethesda, Maryland) analysis software was used to measure the area of CNV, with the operator blinded with respect to treatment groups.

### Flow Cytometry (Analysis and Cell Sorting)

Procedures for cell preparation and staining were as previously reported [19]. Briefly, eyes from at least 5 mice which were laser coagulated or not were extracted and the RPE-choroid complex were gently scraped off the sclera. The RPE-complex was digested with collagenase (Wako, Osaka, Japan), and type II collagenase (Worthington Biochemical Corp., Lakewood, New Jersey) at 37°C. The digested tissue was passed through 40-µm filters to yield single cell suspensions. Cell surface antigen staining was performed as described previously [20]. Anti-CD45, -CD31, -CD11b (Pharmingen, BD Biosciences) mAbs were used for immunofluorescence staining. The stained cells were analyzed and sorted using a FACSAria flow cytometer (BD) with FlowJo (TreeStar) software. Dead cells were excluded from the analyses using the 2D profile of forward versus side scatter.

### Quantitative Reverse-transcription Real-time PCR (qRT-PCR)

For RNA extraction, we used at least 5 mice in each group for sorted cells as indicated above. For analysis of ADM mRNA expression in RPE/choroid complexes, we used 2 mice per day. RNA was extracted from cells using an RNeasy Mini Kit (Qiagen), and cDNA was generated using reverse transcriptase from the ExScript RT reagent Kit (Perfect Real Time) (Takara). Real-time PCR was performed using a Stratagene Mx3000P (Stratagene, La Jolla, CA). Polymerase chain reaction (PCR) was performed on cDNA using specific primers following; mouse CRLR, 5′-ACC TGC ACA CAC TCA TCG TG-3′ and 5′-TGA TCC AGC AAT TGT CGT TG-3′; mouse RAMP2, 5′-CTG AGG ACA GCC TTG TGT CA-3′ and 5′-AAG TCC AGT TGC ACC AGT CC-3′; mouse RAMP3, 5′-AAG GTG GCT GTC TGG AAG TG-3′ and 5′-TGA TGT TGG TCT CCA TCT CG’; mouse ADM, 5′- GAC TCG CTG ATG AGA CGA CA-3′ and 5′- GAA CCC TGG TTC ATG CTC TG’; mouse GAPDH, 5′- TGG CAA AGT GGA GAT TGT TGC C-3′ and 5′- AAG ATG GTG ATG GGC TTC CCG-3′; human ADM, 5′- ATG AAG GGT GCC TCT CGA A-3′ and 5′- CCC TGG AAG TTG TTC ATG C-3′; human GAPDH, 5′- GAA GGT GAA GGT CGG AGT C-3′ and 5′- GAA GAT GGT GAT GGG ATT TC-3′. Expression level of the target gene was normalized to the GAPDH level in each sample.

### In vitro Assays

We examined the in vitro effect of ADM on inflammatory responses of 3 major cell types associated with CNV formation, i.e., microvascular ECs, macrophages, and RPE cells, using the murine cell lines bEnd3, RAW264.7, and the human cell line ARPE-19, respectively. ARPE19 cells were purchased from the American Type Culture Collection (ATCC, Manassas, VA). Cells were cultured in six-well plates for 12 hours in DMEM (Sigma, ATCC and Nikken, respectively). After 4 hours serum starvation using 0.5% fetal bovine serum (FBS), cells were stimulated with tumor necrosis factor (TNF)-α (PeproTech, 50 ng/mL) or lipopolysaccharide (LPS; Wako, 10 µg/mL). After a 12 hour incubation, cell lysate from each line, and the supernatants from cultures of RAW264.7 cells, were processed for real-time RT PCR and EC proliferation assays, respectively. ECs (bEnd3) which were incubated for an additional 4 hours with supernatant plus 1 µM or 5 µM ADM (22–52), or PBS, were quantified using Cell-counting kit-8 (DOJINDO) according to the supplier’s protocol. 1 µM SU1498 was added to the supernatant in some experiments. Dose-response model assessing the toxicity of ADM (22–52) was also performed using same kit after 24 hour incubation of ADM inhibitor at indicated concentration. Absorbance was then measured at 490 nm, and at 630 nm as reference, with a Microplate Reader 550 (Bio-Rad Laboratories). Primary culture of RPE/choroid complexes was performed using cells dissected from 8-week-old wild-type C57BL/6 mice. Tissues were dissociated with trypsin, resuspended in DMEM containing 20% FBS and then plated on laminin-coated 24-well plates at a density of a tissue from1 mouse/well and incubated at 37°C, 5% CO_2_ for 24 h before 4 hours serum starvation with 0.5% FBS. After 12 hours TNF-α treatment, tissues were collected and immediately washed with PBS before RNA extraction. For ELISA, after seeding the primary culture, we cultured for 24 hours as indicated above, then serum-starved for 4 hours in DMEM without FBS before TNF-α stimulation. We stimulated cells with 50 ng/ml TNF-α for 24 hours without FBS. We used an ADM detection kit (Phoenix Pharmaceuticals) according to the supplier’s protocol.

### Immunohistochemistry

The procedure for tissue preparation and staining was as previously reported [21]. For immunohistochemistry, biotin-conjugated anti-CD31 antibody (BD Biosciences, 1∶200) was used for staining and anti-rat IgG Alexa Fluor 488 (Invitrogen, 1∶300) as the secondary antibody. Samples were visualized using DM5500B (Leica) and confocal microscopy (TCS/SP5, Leica). Images were acquired with a DFC 500 digital camera (Leica) and processed with the Leica application suite (Leica) and Adobe Photoshop CS3 software (Adobe systems). All images shown are representative of more than 6 independent experiments.

### Statistical Analysis

All data are presented as mean ± standard error of the mean (SEM). For statistical analysis, the statcel 2 software package (OMS) was used. In experiments involving qRT-PCR and CNV suppression with ADM mAb, we used two-sided Student’s t-test to compare two groups (except for expression of ADM mRNA after CNV induction).We used analysis of variance performed on all data followed by Tukey–Kramer multiple comparison testing in qRT-PCR experiments for expression of ADM mRNA after CNV induction, EC proliferation assays using bEND.3 and for experiments on CNV suppression with ADM and VEGF antagonists. A probability value of less than 0.05 was considered statistically significant.

## Results

**Figure 1:**
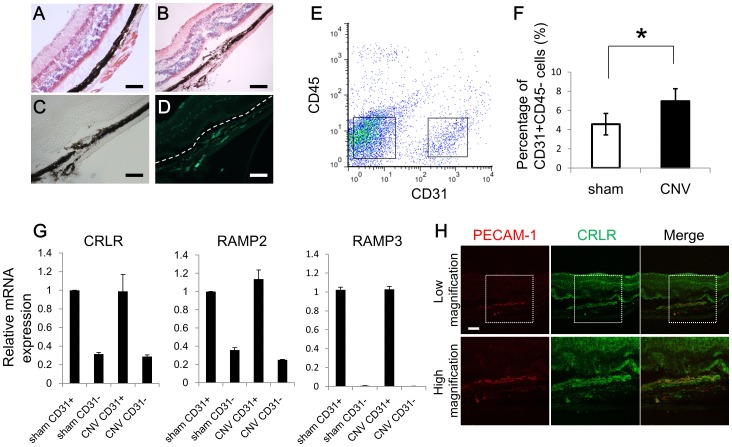
Expression of ADM receptors in choroidal ECs. (A, B) Hematoxylin-eosin-stained light micrograph of normal eye (A) and CNV lesion 7 days after laser treatment (B). Bar, 100 µm. (C) Light micrograph of serial sections of (B). Bar, 100 µm. (D) Immunohistochemistry of (C) with anti-PECAM-1/CD31 antibody. Dotted line indicates the borderline between the neural retina and RPE. Because the CNV lesion did not invade into the retina in this model, we could sort the ECs not from nerural retina but from CNV lesions and choroidal tissue in the following flow cytometry experiments. Bar, 100 µm. (E) Flow cytometric analysis of choroidal ECs from wild-type mice. CD31^+^CD45^−^ cells gated on the right are designated as ECs. (F) Quantitative evaluation of the percentage of choroidal EC 7 days after laser treatment. The number was calculated per cell total (n = ≧5, *P<0.05). (G) qRT–PCR analysis of ADM receptor component expression in choroidal ECs. The value in CD31^+^CD45^−^ECs was compared with that in CD31^−^CD45^−^ non-ECs sorted as gated in (A). Note that the expression level of ADM receptors is not significantly different between ECs and non-ECs in both sham-operated mice (sham) and laser-irradiated CNV mice (CNV). (H) Immunostaining of the CNV 7 days after laser treatment with anti-PECAM-1 (red) and anti-CRLR (green) antibody. High maginification indicate the dotted box in Low magnification. Bar, 100 µm.

To elucidate the involvement of ADM signaling in CNV, we first investigated the expression of the ADM receptor in choroidal vascular ECs in the CNV model. After we confirmed that CNV did not invade retinal tissue (Fig. 1A–D), CD31^+^CD45^−^ ECs in the RPE-choroid complex were analyzed by flow cytometry (Fig. 1E) and their number was calculated as a percentage of a total of 5×10^4^ choroid/RPE complex cells 7 days after laser induction. We confirmed that the number of ECs increases after induction of CNV in laser-treated eyes (6.96% of total cells) compared to control eyes (4.57% of total cells) (Fig. 1F). There was no change in the number of CD31^+^ CD45^+^ or CD31^−^CD45^+^ cells; however, CD11b cells increased after laser-induction of CNV (6.32% vs 1.8%) (data not shown). Because ADM signaling is known to be mediated through CRLR/RAMP2 and CRLR/RAMP3 receptors, we analyzed the expression of CRLR, RAMP2 and RAMP3 mRNA in choroid/RPE complexes after induction of CNV. CRLR, RAMP2 and RAMP3 were highly expressed in the CD31^+^CD45^−^ ECs compared to CD31^−^CD45^−^ non-ECs. However, the level of expression of these ADM receptor components in ECs was not altered after CNV induction compared to sham-operated control groups (Fig. 1G). Moreover, we **c**onfirmed the expression of CRLR in the ECs of CNV by immunostaining (Fig. 1H).

**Figure 2:**
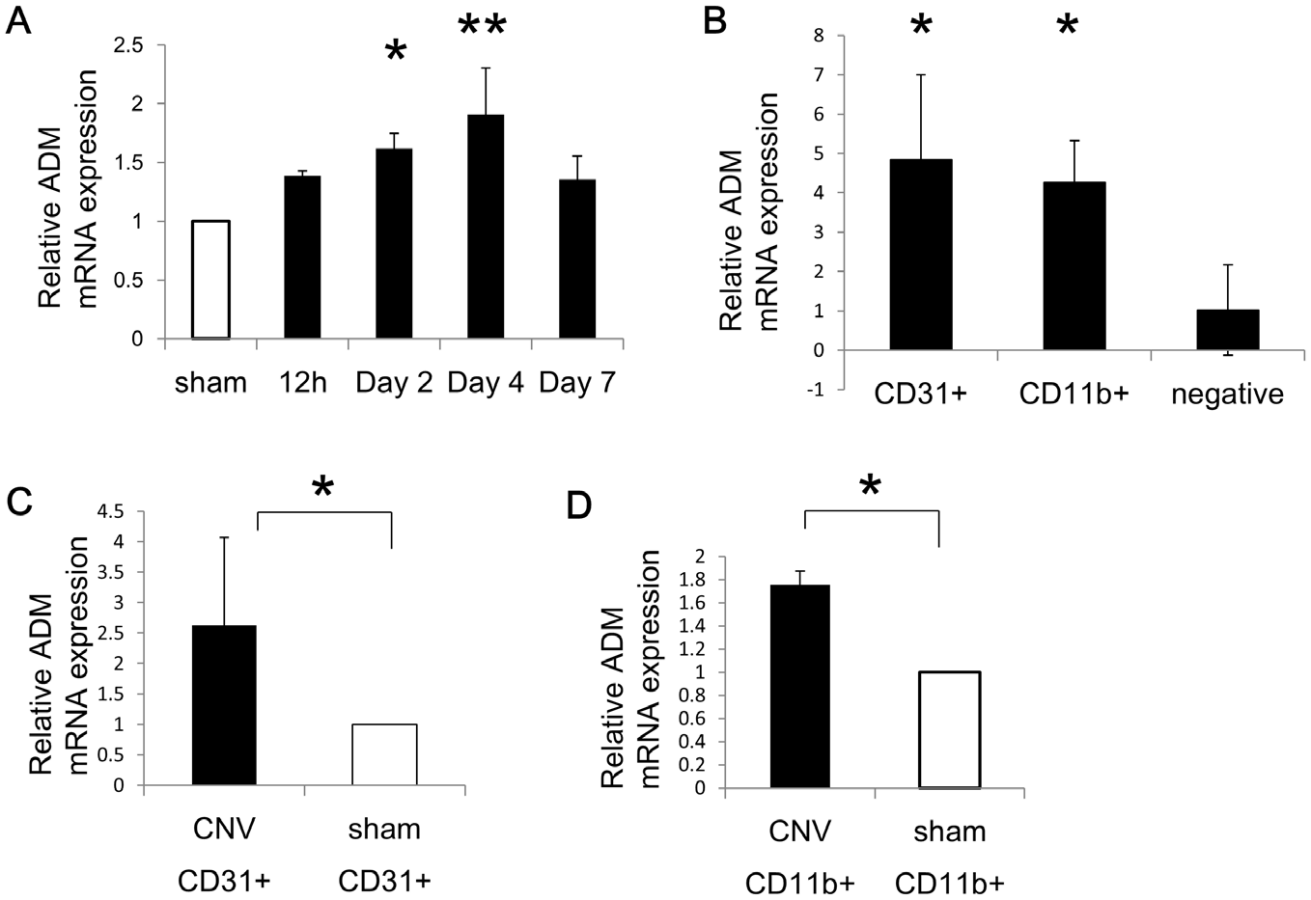
ADM expression after laser treatment. (A) Time course ADM mRNA expression by qRT–PCR analysis in the RPE/choroid complex after laser treatment. Results are shown as fold-increase in comparison with RPE/choroid complexes from sham-operated eyes. (B) qRT–PCR analysis of ADM mRNA expression in sorted CD31^+^ cells (EC-enriched cell population), CD11b^+^ cells (monocyte/macrophage lineage cells) and cells negative for either CD31 or CD11b (non-EC, non-monocyte/macrophage lineage fraction) 3 days after laser treatment (n≧5). (C) qRT–PCR analysis of ADM mRNA expression in sorted CD31^+^ cells after laser treatment (CNV) compared to sham-treated eye**s** (sham). (n≧5, *P<0.05) (D) qRT–PCR analysis of ADM mRNA expression in sorted CD11b^+^ cells after laser treatment compared to sham-treated eyes. (n≧5, *P<0.05).

Next, we assessed the expression of ADM during CNV formation. In the laser-induced CNV model, the presence of different angiogenic factors in RPE-Choroid complexes has already been reported [22]. We analyzed the expression of ADM mRNA and found that it increased in a time-dependent fashion and peaked 4 days after laser treatment (Fig. 2A). Because it is reported that ADM promotes angiogenesis in both an autocrine and a paracrine manner [8], [23], we focused on two cell populations which could be isolated by flow cytometry: CD31^+^ ECs and CD11b^+^ monocytes/macrophages. We confirmed that both ECs and macrophages express more ADM mRNA compared to CD31^−^CD11b^−^ cells (Fig. 2B). Furthermore, after CNV induction, ADM expression was significantly upregulated in both ECs and macrophages compared to the same cells in sham-operated mice (Fig. 2C, D). Therefore, these data suggest that ADM is involved in this laser-induced CNV model.

**Figure 3:**
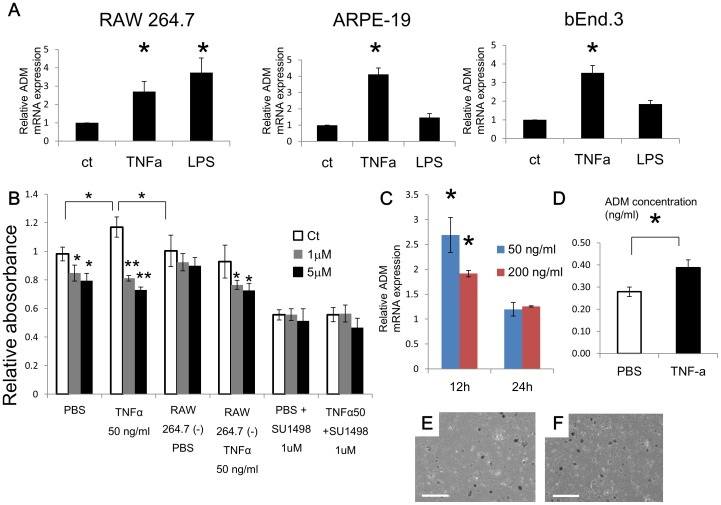
In vitro effect of an ADM antagonist. (A) qRT–PCR analysis of ADM mRNA expression after 12 hr TNF-α or LPS stimulation of cells, as indicated (n = 3, *P<0.01). (B) EC proliferation assay using bEND.3 which was incubated with or without supernatant from TNF-α-stimulated RAW 264.7 in the presence or absence of the ADM antagonist ADM (22–52). The white bar indicates the PBS group, gray indicates the 1 µM ADM (22–52) group and black the 5 µM ADM (22–52) group. ECs in all groups were incubated with supernatant from RAW 264.7 cells except for RAW 264.7 (-) groups (culture medium without supernatant of RAW 264.7 cells). 1 µM SU1498 was added to indicated groups (n = 3, **P<0.01, *P<0.05). (C) qRT–PCR analysis of ADM mRNA expression after 12 and 24 hr TNF-α stimulation of primary RPE/choroid cultures (n = 3, *P<0.05). (D) ELISA of secreted ADM from primary RPE/choroid cultures after stimulation with 50 ng/ml TNF-α or culture with PBS alone (*P<0.05) (E, F) Appearance of primary RPE/choroid cultures after stimulation with PBS (E) or 50 ng/ml TNF-α(F). Scale bar: 100 µm.

It is well known that inflammatory cytokines upregulate ADM expression in various cells [24], [25], [26]. We confirmed this by using macrophage (RAW264.7), EC (bEnd.3), and retinal pigment epithelial (ARPE-19) cell lines stimulated with TNF-α and LPS (Fig. 3A). It has been reported that ADM induces EC proliferation, migration and tube formation through phosphatidylinositol 3′ kinase (PI 3′ -kinase)/Akt, extracellular signal-regulated kinase (ERK), and tyrosine phosphorylation of focal adhesion kinase (p125 FAK) [14]. Thus, we tested whether culture supernatant from RAW264.7 cells after TNF-α stimulation promotes EC proliferation and whether this can be inhibited by a widely-accepted ADM antagonist, ADM (22–52) [27]. Although the inhibitory effect was weak compared to a potent VEGF-A signaling inhibitor, SU1498 [28], ADM (22–52) did significantly suppress proliferation of EC cultured not only in supernatant from TNF-α-stimulated macrophages but also to some extent in medium containing TNF-α without macrophages (Fig. 3B). Additionally, we confirmed the absence of the potential toxicity of ADM (22–52) using an in vitro dose-response model (Fig. S1). These data suggest that ADM signaling affects EC proliferation in an autocrine and a paracrine manner. Moreover, we evaluated the expression of ADM in 24 hour-cultured primary RPE/choroid complexes obtained from wild-type mice. TNF-α stimulation of cultured primary RPE/choroid complexes significantly upregulated ADM mRNA and protein levels at the 12 hour time point (Fig. 3C and 3D); however, expression of ADM mRNA after 24 hours was downregulated relative to the 12 hour time point (Fig. 3C). The morphology of the cells did not change(Fig. 3E and 3F).

**Figure 4:**
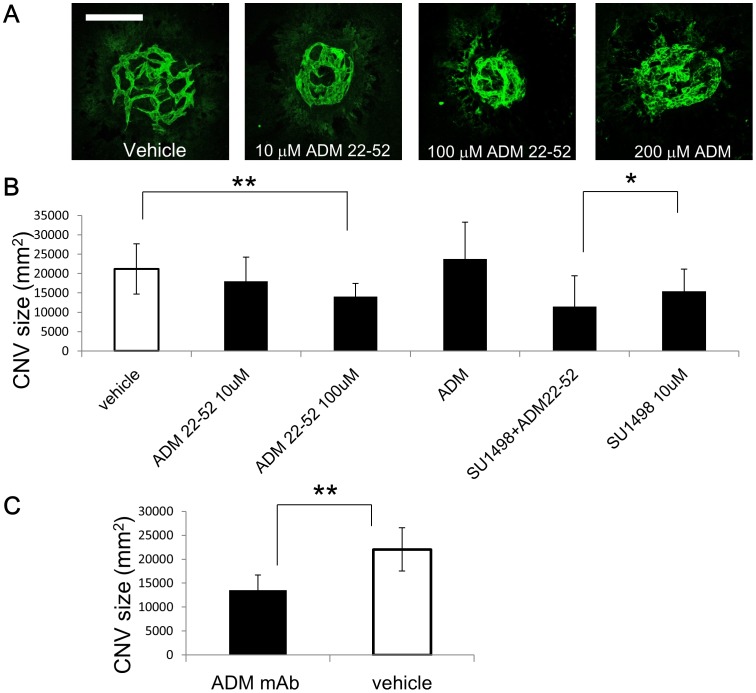
Suppression of CNV in mice by blocking ADM. (A) Flat-mount immunofluorescence staining with anti-PECAM-1/CD31 antibody in the choroid after vehicle, ADM (22–52) or recombinant ADM treatment. Scale bar: 100 µm. (B) Quantitative analysis of CNV size as revealed in (A). (n = 16, **P<0.01) and quantitative analysis of CNV size after treatment with 10 µM SU1498 with or without 100 µM ADM (22–52) (n = 8, *P<0.05). (C) Quantitative analysis of CNV size treated with ADM mAb compared to vehicle. (n = 10, **P<0.01). Error bars indicate mean ± s.d.

To clarify the role of ADM in this CNV model, we inhibit ADM signaling using intraocular injections of ADM antagonist, ADM (22–52). Quantification of CNV size judged by immunohistochemical analysis with anti-PECAM-1 Ab 7 days after laser treatment revealed that intraocular injection of ADM (22–52) significantly inhibited the formation of CNV to approximately 60% of controls. In contrast, injection of recombinant ADM did not affect the size of CNV compared to controls, contrary to our expectations (Fig. 4A and B). This suggests that an excessive amount of ADM is produced in the choroid/RPE in this CNV model. Next, we assessed whether the ADM antagonist had any synergistic effect with SU1498 on CNV. Treatment with ADM antagonist and SU1498 together suppressed CNV formation more than SU1498 alone (Fig. 4C). Furthermore, we confirmed the effect of anti-ADM monoclonal antibody; intraocular injection of ADM mAb resulted in significant suppression of CNV formation (Fig. 4D).

## Discussion

In the current study, we have explored the possibility that ADM exerts angiogenic effects in a laser-induced CNV model. The ADM receptor was expressed in choroidal endothelial cells and ADM was upregulated in the choroid/RPE complex during CNV formation. Moreover, an ADM pharmaceutical antagonist and a mAb to ADM efficiently suppressed CNV formation and mediated synergistic effects together with a potent VEGF-A inhibitor. These findings suggest that ADM could represent a therapeutic target and be an attractive option for the treatment of CNV in AMD.

The biological functions of ADM are well- recognized to be dilatation of resistance vessels [29], [30], increases of cardiac output [31], regulation of vascular permeability [32] and contribution to mobilization, adhesion and differentiation into endothelial progenitor cells of bone marrow-derived cells [33], [34], [35]. Additionally, several lines of evidence have suggested that inhibiting the ADM pathway with antibodies or antagonists directed against ADM or ADM receptors can reduce angiogenesis and tumor cell proliferation in mouse cancer models [8]. Although ADM is reported to affect not only angiogenesis but also tumor cell proliferation via its autocrine and paracrine mechanisms in cancer, there is little data showing the involvement of ADM in a disease model whose central pathogenesis depends on sprouting angiogenesis [11], [12], .

ADM immunoreactivity was reported in cardiac myocytes, vascular smooth muscle cells, ECs, renal distal and collecting tubules, mucosal and glandular epithelia of the digestive, respiratory and reproductive system, the endocrine and neuroendocrine system, as well as in the central nervous system [29]. In the current model, upregulation of ADM was not detected in the retina after laser treatment (data not shown), although Blom et al. reported the expression of ADM in neural retina. However, we saw ADM in the RPE/choroid. Secreted inflammatory cytokines after laser burn might be enhancing ADM expression and further accumulation of inflammatory cells.

This was confirmed using a primary RPE/choroid culture model in the present study. However, ADM expression 24 hr after TNF-α stimulation was lower than at 12 hr, in contrast to findings with different cell lines such as RAW 264.7, RPE-19 and bEnd.3. We hypothesize that this difference can be attributed to the use of transformed cell lines which could display different expression patterns compared to that of primary cultures. Local upregulation of ADM at each photocoagulated site could induce direct ADM effects, i.e. EC proliferation, migration and tube formation. Currently, anti-VEGF therapy has become the major treatment modality for neovascular AMD. However, numerous injections of the anti-VEGF drug may be required to maintain clinical benefit. Moreover, after treatment with anti-VEGF drugs, it can be hypothesized that a hypoxic response could occur and subsequently upregulate ADM, because this has been observed in ECs [25] [36]. Chen et al. reported reciprocal regulation of ADM and HIF-1α expression exerted synergistic effects on proliferation of ECs in vitro [36]. Induction and nuclear translocation of HIF-1 α controls the expression of several angiogenic factors [37]. Therefore, the requirement for additional drugs together with anti-VEGF therapy is rational even if anti-ADM treatment partially suppresses CNV formation.

Although we expected that ADM injection would enhance the angiogenic response, exogenous ADM did not alter the size of CNV. We hypothesize that ADM was already saturated in the lesion and therefore additional factor would not be able to further activate the ADM receptor and exert any greater effects.

Chen et al. reported that tumor-associated macrophages (TAM) express both ADM and ADM receptor components. They also reported that ADM from TAM stimulated ECs and furthered angiogenesis via a paracrine pathway; moreover, it also potentiated the differentiation of TAM from the M1 to M2 state in an autocrine manner [23]. In the current study, infiltrating macrophages in CNV eyes expressed more ADM; therefore we cannot completely exclude the possibility that upregulation of ADM in macrophages could cause them to change their characteristics and to secrete other angiogenic factors such as VEGF. However, the finding of an inhibitory effect on EC proliferation of ADM antagonists in supernatant from macrophage cultures implies that ADM originating from macrophages must be at least partly responsible for CNV formation.

Udono et al. reported that hypoxia and inflammatory cytokines induced the expression of ADM in human RPE cells and that ADM could enhance RPE proliferation [26], [38]. Therefore, in this CNV model, it is also possible that ADM expression in RPE was upregulated and that ADM secreted by RPE could promote angiogenesis. However, Huang et al. reported that ADM inhibited the migration of RPE cells in association with reductions in [Ca^2+^] [39]. Although there is some controversy about the function of ADM in RPE cells, we could demonstrate that inflammatory stimulation up-regulated the expression of ADM in RPE in vitro. Indeed, it is technically difficult to sort the RPE cells by flow cytometry using specific surface markers and we were unable to determine their ADM expression level. Although we have to carefully evaluate the major source of ADM in the CNV model, the observed paracrine and autocrine effects of ADM could induce CNV.

## Supporting Information

Figure S1 **Toxicity experiment of ADM (22–52) using bEnd.3. proliferation assay.** ADM (22–52) could inhibit the proliferation of EC at 10 nM but there was no clear toxicity even at concentration of 100 µM. (*P<0.05).
(TIF)
