# Supplementary figures and images for: An Angiogenic Role for Adrenomedullin in Choroidal Neovascularization
Source: PLoS One. 2013 Mar 8;8(3):e58096. doi: 10.1371/journal.pone.0058096 (PMC3592925; doi:10.1371/journal.pone.0058096)

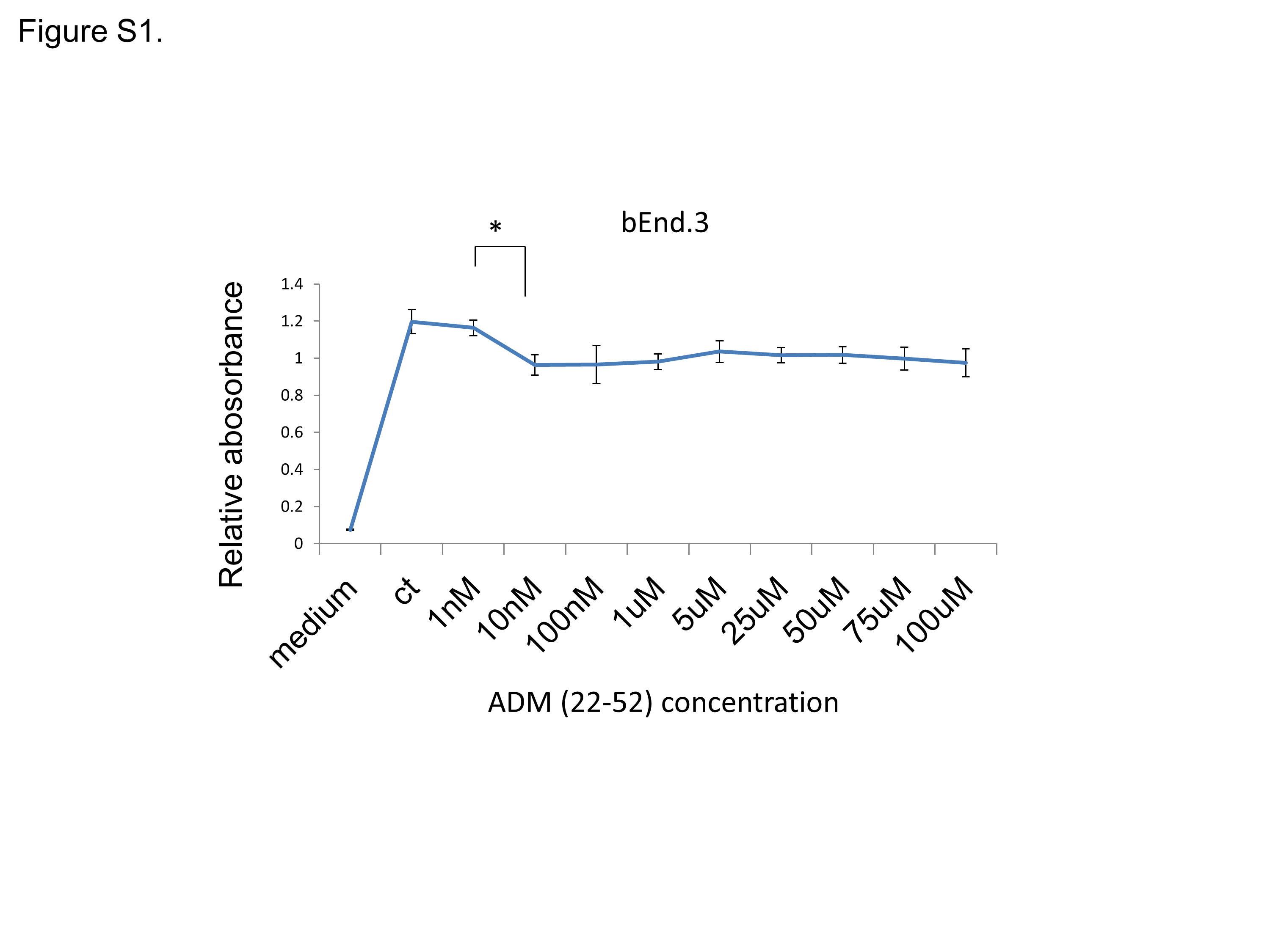

Supplement: Figure S1 — Toxicity experiment of ADM (22–52) using bEnd.3. proliferation assay. ADM (22–52) could inhibit the proliferation of EC at 10 nM but there was no clear toxicity even at concentration of 100 µM. (*P<0.05). (TIF) [file pone.0058096.s001.tif]
